# Silicon Quantum Dots: Synthesis, Encapsulation, and Application in Light-Emitting Diodes

**DOI:** 10.3389/fchem.2020.00191

**Published:** 2020-04-07

**Authors:** Sofia Morozova, Mariya Alikina, Aleksandr Vinogradov, Mario Pagliaro

**Affiliations:** ^1^Laboratory of Inkjet Printing of Functional Materials, SCAMT Institute, ITMO University, Saint-Petersburg, Russia; ^2^Istituto per lo Studio dei Materiali Nanostrutturati, CNR, Palermo, Italy

**Keywords:** silicon quantum dots, synthesis method, fluorescence, quantum yield, microencapsulation, light-emitting diodes

## Abstract

Silicon quantum dots (SiQDs) are semiconductor Si nanoparticles ranging from 1 to 10 nm that hold great applicative potential as optoelectronic devices and fluorescent bio-marking agents due to their ability to fluoresce blue and red light. Their biocompatibility compared to conventional toxic Group II-VI and III-V metal-based quantum dots makes their practical utilization even more attractive to prevent environmental pollution and harm to living organisms. This work focuses on their possible use for light-emitting diode (LED) manufacturing. Summarizing the main achievements over the past few years concerning different Si quantum dot synthetic methods, LED formation and characteristics, and strategies for their stabilization by microencapsulation and modification of their surface by specific ligands, this work aims to provide guidance *en route* to the development of the first stable Si-based light-emitting diodes.

## Introduction

Silicon quantum dots are nanometer-sized particles of crystalline silicon with properties of great interest in light of photonics, microelectronics, and biotechnological applications: high quantum yield (QY, the ratio between the number of photons emitted by a fluorophore and the number of absorbed photons), high lifetime of photoluminescence (PL), wide range of wavelength emission, and non-toxicity (Pavesi and Turan, 2010). Affecting the efficient emission and absorption of light, silicon in the bulk state is a semiconductor with an indirect bandgap. Yet, a decrease in particle size to <5 nm (the excitonic Bohr radius of silicon) allows the conversion of Si particles from indirect to direct bandgap materials with a high quantum yield (QY) of photoluminescence to up to 90% (Li et al., 2016; Gelloz et al., 2019).

In silicon SiQDs of < 5 nm in size, the photoluminescence intensity dramatically increases and blue shifts with further decreasing particle size (Sychugov et al., 2016). As happens with other QDs, the optical and electrical properties of SiQDs can be controlled by varying the particle size, the crystallinity, the nature of the surface groups and the surrounding matrix, and by doping with transition metals (Sychugov et al., 2016).

The methods for producing nanosized silicon particles are rather well-studied and can be divided into chemical and physical approaches. The first include laser ablation (Li et al., 2004; Beard et al., 2007; Vendamani et al., 2015; Xin et al., 2017) and non-thermal plasma synthesis (Cheng et al., 2010; Yasar-Inceoglu et al., 2012; Liu et al., 2016). The second include electrochemical etching (Sato et al., 2009; Castaldo et al., 2014; Chen et al., 2019), reduction of silicon halides (Tilley and Yamamoto, 2006; Cheng et al., 2012; Choi et al., 2014; Sacarescu et al., 2016), thermal destruction of silicon-rich oxides (Hessel et al., 2007, 2011), hydrothermal decomposition of different Si-contained organic precursors (Lopez-Delgado et al., 2017; Liu et al., 2018b; Phan et al., 2018; Yi et al., 2019), oxidation of sodium silicide or Zintl monoclinic phase (Na_4_Si_4_) (Neiner et al., 2006; Atkins et al., 2011; Beekman et al., 2019), mechanochemistry (Chaudhary et al., 2014), processing porous silicon (Gongalsky M. et al., 2019), and others (Holmes et al., 2001; Dasog et al., 2012).

Recent reviews on the utilization of SiQDs in bioimaging and biosensing (Cheng et al., 2014; McVey and Tilley, 2014; Cheng and Guan, 2017; Ji et al., 2018), solar cells (Chen and Yang, 2015), nonlinear optics (Bisadi et al., 2015), and photonics (Priolo et al., 2014; Zhao et al., 2018) show the broad interest and scope of the research concerning these nanomaterials. Less attention has been paid to the features of Si quantum dot-based light-emitting diodes (LEDs), even though in principle one of the most valuable applications of said quantum dots would be in making new LEDs based on abundant silicon in place of current commercial LEDs, which are based on rare earths or on organic phosphors (Buckley et al., 2017).

Compared to organic phosphors, LEDs based on Si QDs are color-pure and photostable and have a narrow emission peak and a wide spectrum of emission controlled by particle size (Cheng et al., 2014). Unfortunately, Si-based LEDs have a very short lifetime. Moreover, the quantum yield is generally low. In general, the monodispersity of silicon QDs is improving lifetime of LED, but quick degradation of LEDs based on nanosized Si occurs due to diffusion and migration of Si atoms to the outer surface of the Si nanoparticle (Maier-Flaig et al., 2013a). Two possible solutions for improving stability have been investigated: microencapsulation of SiQDs or functionalization of their surface. The presence of a shell in encapsulated silicon QDs in which the shell material consists of particles with a larger bandgap, compatible with the lattice of Si QDs, makes it possible to increase the probability of radiative recombination by isolating excitons from surface states (Gong et al., 2015). Another stabilization factor for SiQDs may be encapsulation, the variants of which are considered in the examples of solid dielectric matrices for solar cell applications (Chen and Yang, 2015) and biocompatible polymer matrices for bioimaging (Dasog et al., 2016). Aiming to provide guidelines *en route* to the development of the first stable Si-based light-emitting diodes, in the following, we summarize recent advances in the field of Si-based LEDs, focusing on SiQD synthesis methods affording high (45–90%) QY of photoluminescence, strategies of encapsulation, and recent progress in the formation of LEDs using Si quantum dots.

## SiQD Preparation

**Scheme 1:**
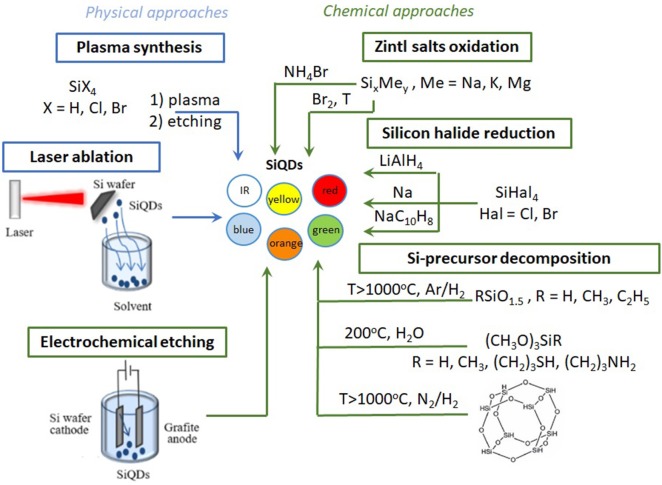
Different approaches to SiQDs synthesis. Blue lines correspond to the physical approaches for SiQD synthesis; green lines correspond to the chemical approaches.

Techniques for SiQD synthesis are divided into physical and chemical methods. Most physical routes are top-down (laser generation, plasma synthesis), with bottom-up methods consisting only of plasma synthesis. Chemical methods include both top-down (decomposition of Si-based precursors, electrochemical etching) and bottom-up approaches (reduction of silicon halides, Zintl phases oxidation). Scheme 1 illustrates the main routes divided into the two main methods.

### Physical Routes to Synthesizing SiQDs

#### Laser Generation

Irradiation of a Si plate with the light of a laser of sufficient power (number of monochromatic photons) results in the formation of QDs of high purity and crystallinity (Li et al., 2004; Beard et al., 2007; Vendamani et al., 2015; Xin et al., 2017). The low monodispersity and low stability of the resulting quantum dots are the main limitations of the method. Nevertheless, it was recently shown that, when extending the femtosecond laser ablation time from 30 to 120 min, the size of the SiQDs formed in 1-octene varied from 4.2 to 1.4 nm, with measured PL quantum yield going from 23.6 to 55.8% (Zhang et al., 2018).

#### Plasma Synthesis

The non-thermal plasma method is used both to obtain Si nanoparticles embedded in thin films and to synthesize free-standing silicon nanoparticles. Hot electrons in a plasma during a microwave discharge lead to the dissociation of precursor molecules such as SiX_4_ (X = H, Cl, Br) (Liu et al., 2016). The advantage of the method is a wide choice of fluorescent color of the nanoparticles obtained. Unlike most bottom-up methods, where only blue-green colors are available, red to orange fluorescence can be obtained using non-thermal plasma synthesis (Cheng et al., 2010; Yasar-Inceoglu et al., 2012). The method requires the utilization of special equipment. Yet, it affords nanocrystals reaching very high luminescence efficiency (QY up to 90%), as lately shown for Si/SiO_2_ core-shell QDs obtained via non-thermal plasma synthesis followed by formation of a thin (~1 nm) oxide shell via high-pressure water vapor annealing (Gelloz et al., 2019).

### Chemical Routes to SiQDs

#### Electrochemical Etching

The electrochemical generation of Si quantum dots makes use of a Si wafer as the cathode and graphite as the anode. The electrolyte generally consists of aqueous HF with H_2_O_2_ or HNO_3_ and different additives (polyoxometalates, for example) (Kang et al., 2007). The technique allows the rapid creation of SiQDs with a range of light of fluorescence from blue to red and with rather narrow size dispersion (Sato et al., 2009; Castaldo et al., 2014; Chen et al., 2019). Originally, the Si QDs thereby obtained were characterized by low QY, but recent advances devoted to surface modification of SiQDs resulted in enhanced QY, up to 55% (Tu et al., 2016).

#### Zintl Salt Oxidation

The reaction of Zintl salts (Me_y_Si_x_, Me = Na, K, Mg, etc.) with silicon halides, gaseous bromine, or ammonium bromide in a boiling solution of glyme or under microwave irradiation affords SiQDs (Neiner et al., 2006; Atkins et al., 2011; Beekman et al., 2019). The advantages of the method are accessibility and scalability, thanks to the use of conventional reagents and equipment that is characteristic of conventional colloidal benchtop chemistry. However, the technique only affords Si nanocrystals fluorescing in the blue-green range of the light spectrum. The luminescence efficiency achieved, in terms of QY, is up to 50% (Bart van Dam et al., 2018).

#### Reduction of Silicon Halides

The reduction of SiCl_4_ using sodium naphthalenide, sodium, lithium aluminum hydride, or tetraethylorthosilicate as a reducing agent quickly produces Si nanocrystals (Cheng et al., 2012; Choi et al., 2014; Sacarescu et al., 2016). As expected, however, the particle size distribution is very wide. The addition of surfactant molecules to create micelle “nanoreactors” gives some control over the size (Tilley and Yamamoto, 2006). Usually, this method only gives blue luminescent nanocrystals of colloidal silicon, but high QY up to 90% can be obtained (Li et al., 2016).

#### Decomposition of Si-Containing Precursors

The hydrothermal decomposition of organosilicates such as N-[3-(trimethoxysilyl)propyl]-ethylenediamine (DAMO), 3-aminopropyl triethoxysilane (APTES), or 3-aminopropyl trimethoxysilane (APTMS), in the presence of reducing agents such as LiAlH_4_, sodium citrate, NaBH_4_, and thiourea affords Si QDs (Dasog et al., 2012; Lopez-Delgado et al., 2017; Liu et al., 2018b; Phan et al., 2018; Yi et al., 2019). By varying the reaction time, temperature, and the nature of reducing agent and of the precursor, nanocrystals with QY 65–85% can be synthesized (Ma et al., 2018; Abdelhameed et al., 2019). It is also possible to synthesize SiQDs by the thermal decomposition either of silicon monoxide (SiO) powder heated to 1,350°C (Lu et al., 2017) or of other precursors, such as silsequioxanes, followed by etching and hydrosilylation (Yu et al., 2017).

#### Template Synthesis

SiQDs could be obtained in gram-scale quantity by metallothermal reduction. In one approach, mesoporous SiO_2_ obtained via template-assisted sol-gel synthesis is reduced using magnesium powder at 500°C to yield silicon nanocrystals that are reacted with trioctylphosphine oxide to yield hydroxyl-terminated, encapsulated Si QDs exhibiting red luminescence (Dasog et al., 2012). Free-standing NCs liberated using HF acid and further functionalized with alkyl groups yield NCs that are dispersible in organic solvents with QY up to 48% (Kirshenbaum et al., 2018).

Data concerning the state of the art in SiQDs synthesis with respect to luminescence efficiency (QY of PL 45–90%), wavelength of emission, average nanoparticle size, and surface group are summarized in Table 1A.

**Table 1:** Main features of SiQDs obtained via different synthetic routes and the main recent advances in SiQD LEDs.

**Synthesis technique**		**PL wavelength, nm**	**Particle size, nm (size distribution, %)**	**Surface group**	**QY, %**	**References**
**(A) MAIN FEATURES OF SiQDs OBTAINED VIA DIFFERENT SYNTHETIC ROUTES**						
Physical	Laser generation	430	1–2 (80%)	1-octene	55.8	Zhang et al., 2018
	Non-thermal plasma synthesis	825	4	Si/SiO_2_	90	Gelloz et al., 2019
Chemical methods	Electrochemical etching	621	5-8	Complex shell^*^	55	Tu et al., 2016
	Zintl salt oxidation	650	2.2	n-butyl	50^**^	Bart van Dam et al., 2018
	Reduction of silicon halides	520	4.5–6 (60%)	1,2,3,4-tetrahydrocarbazol-4-one	90	Li et al., 2016
	Hydrothermal decomposition DAMO^***^	445	4–5	Citrate/thiourea	73.3	Ma et al., 2018
	Hydrothermal decomposition DAPTMS^***^	445	4.1	Citrate/thiourea	84.9	Ma et al., 2018
	Hydrothermal decomposition APTES^***^	515	1–3 (97%)	Fluorescein isothiocyanate	64.7	Abdelhameed et al., 2019
	Decomposition of silsesquioxane	400	6.1	Dodecene and SiO_2_ matrice	45	Yu et al., 2017
	Template synthesis	645-712	2.9–3.6	dodecyl	48	Kirshenbaum et al., 2018
**SiQD synthesis technique**	PL, wl^****^, nm (QY, %)	EQE^*****^, %	L^******^, Cd/m^**2**^	**V**_T_^*******^, **V**	LED structure ^********^	**References**
**(B) The MAIN RECENT ADVANCES IN SiQD LEDs**						
TES decomposition and encapsulation in SiO_2_	620	0.033	4,200	2.8	ITO/ZnO/SiQDs/CBP/MoO_3_/Al	Yamada and Shirahata, 2019
TES decomposition	710 (25)	0.035	–	3.5	ITO/PEDOT:PSS/polyTPD/SiQDs/ZnO/Al	Ghosh et al., 2014
(HSiO_1.5_)_n_ thermal decomposition	625–680 (43)	0.09–0.074	22.6	1.8	glass/TPBi/SiQDs/polyTPD/PEDOT/ITO/glass	Maier-Flaig et al., 2013b
TES decomposition	720–840 (44–56)	0.20–0.23	4.4–5.5	2.5–2.1	ITO/PEDOT:PSS/SiQDs/TPBi/Al	Ghosh et al., 2018a
Non-thermal plasma synthesis	740 (31)	2.4	–	6.4	ITO/PEDOT:PSS/polyTPD/SiQDs/ZnO/Ag	Gu et al., 2017
Non-thermal plasma synthesis	700 (40)	2.7	–	6.0	glass/ITO/PEI/ZnO/SiQDs/TAPC/MoO_3_/Al	Yao et al., 2016
TES decomposition	720 (40)	3.1	5,000	3.5	ITO/PEDOT:PSS/polyTPD/SiQDs/TPBi/Al	Ghosh et al., 2018b
Non-thermal plasma synthesis	735 (47)	6.2	–	–	ITO/PEDOT:PSS/polyTPD/PVK/SiQDs/ZnO/Ag	Liu et al., 2018a
Non-thermal plasma synthesis	777 (43)	8.6	–	1.3	ITO/PEDOT:PSS/polyTPD/SiQDs/CPB/LiF,Al	Cheng et al., 2011

* SiQDs - (ethylenedioxy)diethanethiol-1-(2-isothiocyanatoethyl)-1H-pyrrole-2,5-dion/bovine serum albumin/ isothiocyanate–PEG–isothiocyanate/antibode;

** -internal quantum efficiency;

*** DAMO – N-[3-(trimethoxysilyl)propyl]ethylenediamine; DAPTMS – [3-(2-aminoethylamino)propyl]trimethoxysilane; APTES – 3-aminopropyl triethoxysilane;

**** PL-photoluminescence, wl-wavelength;

***** EQE-external quantum efficiency;

****** L-luminance;

******* V_T_-turn-on voltage;

******** *TES is triethoxysilane; CBP is 4,4′-Bis(N-carbazolyl)-1,1′-biphenyl; TPBi is 2,2',2”-(1,3,5-benzinetriyl)-tris(1-phenyl-1-H-benzimidazole); polyTPD is poly(bis-4-butylphenyl-N,N-bisphenyl) benzidine; PEDOT:PSS is poly(3,4-ethylenedioxythiophene) polystyrene sulfonate; PEI is polyethyleneimine; TAPC is 1,1-bis[(di-4-tolylamino)phenyl]cyclohexane; PVK is poly(9-vinlycarbazole)*.

## Size Separation of SiQDs

Size separation methods of SiQDs are important because precise monodispersity is required in many applications, including LED devices (Maier-Flaig et al., 2013a), due to the dependence of luminescence on the nanocrystal size and size distribution, influencing the purity of the emitted color and the quantum yield. Several techniques can be applied for size-separation of QDs: field flow fractionation, membrane methods, and size exclusion chromatography (Mori, 2015). Field flow fractionation is directed by variant force fields: crossflow stream, temperature gradient, electrical potential gradient, centrifugal, dielectrophoretic, and magnetic forces. However, the application of an electric or magnetic field imposes restrictions on the particles used, since they must contain ionic/dipole fragments or have magnetic properties (Mastronardi et al., 2012). Ultracentrifugation and size-selective precipitation are the most common and scalable approaches to produce SiQDs with polydispersity index <1.01 (Rinck et al., 2015; Brown et al., 2017).

## Microencapsulation of SiQDs

The application of SiQDs requires their chemical and physical stabilization (Maier-Flaig et al., 2013a; Buckley et al., 2017). Modern stabilization techniques include ligand exchange (Purkait et al., 2016) and microencapsulation in inorganic (Chen and Yang, 2015) or organic (polymer) (Dasog et al., 2016) matrixes. Organosilicon polymers, in particular, are promising candidates for encapsulation due to their affinity to SiQDs, high transparency in the visible region, and high thermal and photostability (Pagliaro, 2009; Vinogradov and Vinogradov, 2014).

### Hydrolysis and Polycondensation of Trichlorosilane and Methyl Trichlorosilane

The first attempt at the encapsulation of SiQDs involved thermal processing of hydrogen silsesquioxane SiQD precursor in 5% H_2_/95%Ar in the presence of a silica matrix (HSiO_1.5_)_*n*_ or of a methyl-modified silica matrix [(HSiO_1.5_)_*n*_(CH_3_SiO_1.5_)_*m*_], in turn obtained from hydrolytic polycondensation of HSiCl_3_ or of HSiCl_3_ and (CH_3_)_3_SiCl (Henderson et al., 2009). An important finding was that greater networking and cross-linking density of (HSiO_1.5_)_*n*_, resulted in the formation of smaller SiQDs in comparison to HSQ, whereas increasing the amount of methyl groups in the organically modified silica (ORMOSIL) produced larger Si nanocrystals (Palmisano et al., 2006).

### Encapsulation in Mesoporous Silica

A straightforward approach lately demonstrated to increase the water dispersibility and photostability of SiQDs requires encapsulation into mesoporous silica through a simple condensation reaction in which mesoporous silica dispersed in toluene is mixed with a SiQD solution in C_2_H_5_OH, followed by heating to 110°C for reflux for 3 h. The resulting encapsulated SiQD has excellent hydrophilicity, good biocompatibility, low cytotoxicity, retains a high surface area, and exhibits better fluorescence stability in acidic solutions, making it ideally suited for biological applications (Huang et al., 2018; Phatvej et al., 2019). A procedure for obtaining SiQDs capped with an Si–C bonded alkyl layer by heating in an ultrahigh vacuum at 200°C has been reported (Chao et al., 2007). This type of encapsulation may be useful for the controlled preparation of new quantum-confined silicon structures and could facilitate their mass spectroscopic study (Gongalsky M. B. et al., 2019).

## Application of SiQDs in Led Devices

The main characteristics of LEDs are the turn-on voltage (*V*_T_), which characterizes the beginning of working of the device, luminance (*L*), which describes the brightness of the device, and external quantum efficiency (EQE, Equation 1), which is associated with the efficiency of the diode (Ghosh et al., 2018a):

(1) $$\begin{array}{l} EQE\;\left(\%\right)=\frac{q\times P}{J\times E}\times 100\% \end{array}$$

where *q* is the electron charge, *P* is the optical power density, *J* is the current density, and *E* is the energy of a photon emitted by the LED. Table 1B summarizes recent advances in SiQD LEDs producing light-emitting diodes with the highest EQEs. Table 1 also includes a description of the LED design. We briefly remind the reader that design generally involves one electrode being made from indium tin oxide (ITO) glass and the other from aluminum or silver. The multilayer structure of a typical LED consists of an electron injection layer, an electron transfer layer, an optically active layer, a hole transfer layer, and a hole injection layer (Khriachtchev, 2016). Nanoparticles of ZnO are usually used as the material for the electron transfer layer (Yang et al., 2016; Kim and Park, 2017), whereas MoO_3_, WO_3_ are often used in the hole transfer layer due to the simplicity of their synthesis (Son et al., 2009; Wang et al., 2014). The best values of EQE for SiLED are 8.6% for near-infrared emission (Cheng et al., 2011), while for white, red, and orange emissions, values of EQE are less than 0.1% (Maier-Flaig et al., 2013b; Yamada and Shirahata, 2019). There are several ways of improving LED characteristics, including using a device with an inverted structure of instead of a direct one (Yao et al., 2016; Ghosh et al., 2018b; Yamada and Shirahata, 2019), the control of the thickness of the SiQDs and polyTPD layers (Ghosh et al., 2014), the control of the size-dependence of SiQDs (Maier-Flaig et al., 2013a), and using aromatic ligands on SiQD surfaces instead of aliphatic ones (Liu et al., 2018a).

## Conclusions and Perspectives

Significant progress has been made in the synthesis of Si quantum dots with high quantum yield. The development of light-emitting diodes based on SiQDs has so far been limited by their short lifetime, ranging from several hours (Gu et al., 2017; Liu et al., 2018a) to several days (Maier-Flaig et al., 2013b). Fundamental recent works aimed at investigating the origins of such low lifetimes have shown that the destruction of the diode is associated with the migration and diffusion of nanoparticles, as well as with the appearance of macro- and microscopic surface defects on the layers (Maier-Flaig et al., 2013a). Monodisperse SiQDs, in any case, are less prone to migration, allowing for a considerably longer lifetime of the device. Furthermore, significant progress in nanomaterial synthesis, translating into photoluminescence quantum yield of up to 90%, has not been matched in electroluminescence, as Si-based LEDs generally achieve QY of up to 47%. Intense research efforts are currently being aimed at developing SiQDs with high QY and external quantum efficiency and long lifetime. Along with the use of monodisperse Si nanoparticles, their microencapsulation holds promise for the first practical applications. While, for certain photonics technologies such as photoluminescence-based sensing in biology, encapsulation of SiQDs in mesoporous silica particles is already suitable for practical uses, this is not yet the case for LEDs. Due to the low cost, large abundance, and excellent health and environmental profile of silicon, when and if the issue of their poor stability will be solved, the use of silicon quantum dots will develop to become the dominant technology in photonics.

## Author Contributions

MA and SM organized and wrote the manuscript. AV and MP discussed the results. All authors approved this manuscript.

### Conflict of Interest

The authors declare that the research was conducted in the absence of any commercial or financial relationships that could be construed as a potential conflict of interest.
